# Why do drivers become safer over the first three months of driving? A longitudinal qualitative study

**DOI:** 10.1016/j.aap.2018.04.007

**Published:** 2018-08

**Authors:** Marianne R. Day, Andrew R. Thompson, Damian R. Poulter, Christopher B. Stride, Richard Rowe

**Affiliations:** a Department of Psychology, University of Sheffield, UK; b Department of Psychology, Social Work and Counselling, University of Greenwich, UK; c Management School, University of Sheffield, UK

**Keywords:** Automobile driving, Risk-taking, Young adults, Novice drivers, Qualitative, Longitudinal

## Abstract

Drivers are at high crash risk when they begin independent driving, with liability decreasing steeply over the first three months. Their behavioural development, and other changes underlying improved safety are not well understood. We adopted an innovative longitudinal qualitative design, with thirteen newly qualified drivers completing a total of 36 semi-structured interviews, one, two and three months after acquiring a full UK driving license. The interviews probed high-risk factors for new drivers, as well as allowing space for generating novel road safety issues. Analysis adopted a dual deductive and inductive interpretative thematic approach, identifying three super-ordinate themes: (1) Improvements in car control skills and situation awareness; (2) A reduction in the thrill of taking risks when driving against a background of generally increasing driving speed; (3) Early concerns about their social status in the eyes of other road users during the early stages of driving, which may put pressure on them to drive faster than they felt comfortable with. The study provides important new leads towards understanding how novice driving becomes safer over the first few months of driving, including how well-studied concepts of driving skill and style may change during development of independent driving, and bringing the less rigorously studied concept of social status into focus.

## Highlights

• Newly qualified drivers’ crash risk declines over the first three months of independent driving.
• The current study aimed to better understand the factors underpinning this safety improvement.
• A longitudinal qualitative design identified possible contributing factors to this decline in crash risk.
• Developments in skill, thrill-seeking and feelings of driving status were reported.
• Implications for research and application are discussed.

## 1 Introduction

Road traffic crashes are one of top ten global causes of mortality resulting in approximately 3400 deaths per day (Peden et al., 2004; World Health Organisation, 2013). Younger or novice drivers are at greater risk than older or experienced drivers. Studies of novices who began driving at different ages indicate that age and experience have independent effects on crash risk, with some evidence that the effect of experience is greater than that of age (McCartt et al., 2009). Experience is a particularly important protective factor in the early months of independent driving; crash risk declines steeply over this period, irrespective of the driver’s age when obtaining a license (McCartt et al., 2009). However, the behavioural changes that underpin this reduction in crash risk are unclear.

Identifying the behavioural developments that underpin this fall in crash liability over the first few months of driving would inform efforts to improve novice driver safety. Pre-driving interventions usually result in null or limited safety benefits (Glendon et al., 2014; Poulter and McKenna, 2010; Roberts and Kwan, 2006). In contrast, educational interventions that have targeted intentions towards health behaviours such as smoking, drinking, safe sex, and exercise have led to safer behavioural outcomes (Webb and Sheeran, 2006). Therefore, it seems plausible that pre-driving education programs could improve road safety if they adopt effective behaviour change techniques and, crucially, focus on the key behaviours involved in novice driver safety. One approach might aim to equip pre-drivers with the safer driving behaviours that otherwise naturally develop only during the first few months of independent motoring.

Many existing driving behaviour measures, predict crash involvement in novice drivers (de Winter et al., 2015; Horswill et al., 2015) but do not appear to capture the key elements that underlie the improvement in road safety over the early months of driving. Current approaches differentiate between driving skill and style (Elander et al., 1993). Skill includes perceptual-motor skills such as steering and gear-changing. General models of skill development propose that perceptual-motor performance becomes faster and more automatic with practice, making fewer demands on attentional resources (Logan, 1988). There is evidence that self-reported driving errors become more common over the first three years of driving (Roman et al., 2015). It is possible that increasing error-rate might indicate the development of automaticity, as attentional slips and lapses are more likely in the performance of well-practiced tasks which only require minimal attentional input (Reason, 1990). We do not know of any studies that directly test the extent to which car control skills become automatic during the learning period or continue to develop post-licensure.

Driving skill also involves the processes underlying situation awareness. Situation awareness is defined as “the perception of the elements in the environment within a volume of time and space, the comprehension of their meaning, and the projection of their status in the near future” (Endsley, 1995, p. 36). In driving, situation awareness is often measured through hazard perception video simulations which measure the ability to anticipate dangerous traffic situations (Horswill and McKenna, 2004). Hazard perception is related to experience when measured in years (e.g., Wallis and Horswill, 2007) but as yet there is only limited research addressing development over the first few months. One small scale study found no substantial differences in hazard perception measured 1, 5 and 9 months post-licensing (Sagberg and Bjornskau, 2006).

Driving style refers to deliberate choices in terms of speed, following distance and engagement in other violations of recognized safe driving practices. A number of studies indicate that violations become more common in the early stages of driving (Ozkan et al., 2006; Roman et al., 2015; Rowe et al., 2013). This is a counter intuitive finding given the well-established associations between crash involvement and driving violations (de Winter et al., 2015; Evans, 2004).

One possibility is that the measures being used in the studies reviewed above, whilst successful in predicting crash risk in novices, are not sufficiently nuanced to identify the precise behaviours that become safer in the early stages of driving. For example, while driving speeds become faster overall, there may be particularly high risk situations in which novice drivers learn that speed reduction is paramount to safe driving. These might include driving around bends and driving at night; both high crash risk situations for inexperienced drivers (Clarke et al., 2006). Therefore, new behavioural tools may be required to provide more fine-grained assessment of the key behaviour changes that underlie the improvement in driving safety over the first few months of driving. The Behaviour of Young Novice Drive Scale (BYNDS; Scott-Parker and Proffitt, 2015) was constructed from the literature on young drivers to measure relevant aspects of skill, style and exposure to risky situations, including driving at night and driving with same age peers. The BYNDS has five subscales, including one measuring transient violations (that can change across a journey, such as speed choice), and one measuring fixed violations (that are unlikely to change across a journey, such as wearing a seatbelt) as well as a scale measuring exposure to risky situations, including driving at night and driving with same age peers. In a New Zealand study, the exposure to risky situations scale was independently associated with self-reported crash involvement (Scott-Parker and Proffitt, 2015). Data are so far unavailable on whether BYNDS scores change over the first few months of driving.

This study took a fresh approach to examining the behavioural development of new drivers by using a detailed qualitative investigation. Qualitative methods have rarely been employed in driving behaviour research. Exceptions include the use of individual and small group interviews with young and novice drivers about normative influences on risky behaviour (Scott-Parker et al., 2012), and focus group research on young drivers’ perceptions of early driving, including the perceived importance of gaining a sufficient quantity and variety of experience soon after passing the driving test (Glendon, 2013), perceptions of risk and vehicle handling competency among young rural drivers (Knight et al., 2012), and a study of social influences on speed choice (Fleiter et al., 2010). The latter study highlighted that drivers feel pressure from other motorists to drive faster, an effect that has received little attention in quantitative studies. Ehsani et al. (2015) have also employed qualitative methods to explore the perceptions of young drivers on the implications of driving with passengers of similar age, finding that they are aware of the direct and indirect influences on their behaviour.

To date there have been no qualitative studies that have sought to gain repeated information as driving experience develops. Uniquely in the novice driver literature, we used a longitudinal qualitative design in which drivers were interviewed at approximately 1 month, 2 months and 3 months after acquiring a full UK driving license that qualifies then to drive independently. This approach facilitated reflection upon driving development over time. A dual deductive and inductive interpretative thematic analytic approach was adopted (Joffe, 2012). This enabled both the close examination of existing theory/knowledge, whilst allowing novel concepts to emerge. As such, our semi-structured interviews targeted behavioural change in situations in which novice driver crashes commonly occur and become less frequent with experience as identified in a study of 3000 crashes involving UK young drivers (Clarke et al., 2006). These situations included driving around bends, following distance (relevant to rear-end shunts), driving at night and turning right at junctions (i.e., across the oncoming traffic flow, equivalent to a left turn in countries that drive on the right). We also probed for development in speed choice. This has been shown to be a robust predictor of crash involvement (Evans, 2004), and a desire to drive faster may underlie many other forms of dangerous driving. Probes asking participants to generate other areas of challenge and improvement provided space for novel aspects of safer driving over the early months to emerge.

## 2 Method

### 2.1 Participants

Thirteen newly qualified drivers (aged 17–19 years, 6 male, 7 female) who had passed their test within one month of their first interview were recruited through educational establishments and driving instructors in the North of England. This age and experience range was selected as being representative of young drivers at high crash risk (Williams and Carsten, 1989). All were White British and in full-time education. Nine owned cars and four had regular access to a car. Five participants had a telematics device fitted to monitor their driving as part of their insurance policy at first interview, and another participant had a device fitted during the study. Ten had passed their first driving test, two passed on their second attempt, and one passed on the third attempt. Seven participants drove 5–7 days a week, four drove 3-5 days, one drove 1-2 days a week, and one drove less often. None of the participants had received any traffic citations and none had been involved in a crash while driving. All participants provided informed consent. The study procedures were approved by the Research Ethics Committee of the Department of Psychology, University of Sheffield.

### 2.2 Data collection

A total of 36 interviews were conducted; all but three participants took part in all three stages of data collection. The number of participants and amount of interview data generated is well within the range suggested as being sufficient for saturation to be achieved (Guest et al., 2006). Individual semi-structured interviews were carried out by MRD, either in the participants’ educational establishment or home. The interview schedule [supplementary materials] covered known risky driving situations for novices; speed, cornering, right turns, night driving, close following and general driving behaviour. The schedule was devised with an awareness of the literature on risk factors among novice and young drivers (e.g., Clarke et al., 2006), as well as discussion with experts in the field. The interviewees were asked to describe their behaviour in each area and to describe changes over the previous month. They were also asked to describe other aspects of driving that they had found challenging during the previous month, and how they thought their driving had changed.

When driving changes were described, the questioning followed a critical incident technique (Flanagan, 1954; Hughes et al., 2007). This involved asking the interviewee to describe the cause and outcome of a critical incident, their feelings and perceptions of the situation, the actions they took during the incident and any changes in their consequent behaviour. Identical questions were asked at each interview to ensure that responses were comparable. Notes from previous interviews were available at follow-ups and participants were asked whether points made at earlier contacts were still valid. Within the schedule structure the interviews aimed to be conversational with interviewees choosing the order of topic discussion. Interviews lasted between 20 and 47 min and were audio-recorded and transcribed verbatim.

### 2.3 Data analysis

Thematic analysis was used to identify patterns of meaning in the interviews and transform the data into codes and themes (Joffe, 2012). NVivo 10 (QSR International, 2012) was adopted for initial coding and theme construction. A hybrid method (Fereday and Muir-Cochrane, 2008) was used to include both deductive and inductive codes in describing and summarizing the data. The interview probes addressing problematic areas (e.g., cornering) meant that these codes were automatically present in the data. Other codes emerged during data analysis.

Initial analysis involved reading the set of interviews from each participant to obtain a sense of change over time. Memos were added to the data to identify ‘codable moments’ of behavioural development in the transcripts (Boyatzis, 1998) and were summarized to construct a coding frame. This frame formed a template (Crabtree and Miller, 1999) and data from each participant was added into the frame until it was saturated. Saturation was reached when all data was codable under the existing framework, and no new codes appeared to be emerging (Guest et al., 2006). The coding frame and initial coding of three transcripts was carried out by MRD and audited by ART (Spencer and Richie, 2012). Following Boyatzis (1998), a code book and code tree were written to define each code with a description, exclusions and examples of inclusions. All transcripts were then fully coded accordingly. Initial codes were organized into superordinate themes and subthemes with common patterns of meaning, with consideration for relationships between themes and codes. Tables of codes and themes were constructed to map transcripts (participant and time point) onto themes, highlighting common and individual improvements and challenges in driving across the sample.

## 3 Results

Our analysis identified three super-ordinate themes; (1) Driving skills, (2) violations and thrill-seeking, and (3) social status and pressure. Within driving skills, we identified sub-themes addressing control skills and situation awareness.

### 3.1 Driving skills

#### 3.1.1 Control skills

In the earliest stages of driving most respondents reported some difficulties with car control skills such as gear changing, steering and road positioning. Many worried that they were likely to stall,^1^ and also reported difficulties driving between parked cars and negotiating narrow country roads. Therefore, they adopted what they perceived as an over-cautious approach to driving:

“I’m struggling with the idea of ‘will I stall it in their path’, so I’d rather just wait for a gap where there’s no cars coming at all and I can’t hinder people if I do stall it, erm so I will just sit there for massive gaps (laughs).” Participant 1 Interview 1

“And when there’s parked cars on both sides, I struggled with that. I used to want to stop and cause loads of traffic behind me, because I daren’t go through. I’m a lot better at that.” Participant 6 Interview 3

Spatial awareness was a particular difficulty in night driving on unlit roads, a scenario in which a few drivers reported finding it difficult to judge both the dimensions of other cars and the position of their own and other cars on the road:

“…I always then think I’m bigger and the spaces are narrower cos there’s all these different lights…” Participant 13 Interview 1

Seven drivers said that getting used to a different car after passing their test set back their driving skills. These drivers reported that changing cars negatively impacted on control skills such as gear changes, clutch control and steering, and that they felt they needed to ‘relearn’ a feel for these driving techniques:

“I was like, oh God, this is like learning to drive again…the bite point is in a completely different place and everything, so you’re as hesitant as you were when you started to learn.” Participant 8 Interview 3

All of the participants reported that car control skills and an implicit feel for their car’s spatial dimensions and dynamic capabilities improved with practice during the first two months of driving. They reported that they could control the car without consciously thinking about it, could pull away more smoothly, stopped worrying about stalling, and were able to judge the spaces they could fit into. These perceived improvements were positively associated with driving confidence and acceptance of shorter following distances, and smaller gap acceptance at junctions and roundabouts.

“… it's just a natural thing now, it’s like walking, I can get in a car and just drive…” Participant 9 Interview 2

This maturation process included an element of trial and error. Participants reported pulling out when they later felt they should not have done:

“…at the start I was more likely to wait, and then probably last month I’d have pulled out and thought that was a bit close and then carried on, this month I’m probably more timing better.” Participant 7 Interview 3

Having to make their own judgements and have confidence in their decisions was described as an important part of their driving skill acquisition. Although new drivers had to make these judgements while they were learning, the supportive presence of an instructor made these judgements feel qualitatively different:

“Yes I think it's learning the car and just being out by yourself and having to make all the decisions and learning when to go and when to wait and that.” Participant 4 Interview 1

#### 3.1.2 Situation awareness

The participants reported difficulties with understanding the road situation in the early weeks of driving unaccompanied. Some drivers described being unsure where to look at junctions and roundabouts, and struggling in situations where many things were happening at once; such as when there was busy traffic, multiple hazards (e.g., pedestrians, cyclists) or reduced visibility. Some felt their ability to focus on the external road situation was constrained by the attentional demands of car control. They recounted difficulties in driving in novel environments and worried about their ability to react quickly in hazardous situations. Therefore, in the early stages, many drivers favoured familiar routes where they had prior experience of corners, speed limits, road layouts, and routes.

“…it’s not really the roads that’s scary, it’s just the other cars, because you just don’t know what they’re going to do.” Participant 6 Interview 1

With experience, participants reported being more able to focus on the wider road situation, generalize between driving contexts and felt that they could anticipate further ahead, including predicting the actions of other drivers without conscious awareness of the cues they were using.

“I feel like when you become a driver, you end up being able to sense what the other driver's going to do, especially at like roundabouts and stuff.” Participant 11 Interview 2

### 3.2 Violations and thrill-seeking

Many of the participants believed their lack of competence limited their speed during the early weeks. All participants reported driving increasingly fast over the study duration, which they attributed to improved driving skills and confidence. They drove closer to other cars, took corners more quickly and accepted smaller gaps at junctions. A few drivers tested how fast they could control their cars around corners or tried to drive up to the speed limit on faster roads. For some drivers, this increase in speed demonstrated that their driving skills had improved although it also led to some mistakes, for example on cornering:

“…I've gone round corners in the (place name) and I've swung out to the other side of the road and I've thought quite lucky there wasn't a car there…” Participant 5 Interview 1

A few of the participants reported that they sometimes drove aggressively. They had driven closely behind someone they felt was driving too slowly or had cut in front of them; or had become annoyed at people speeding up when they were trying to overtake. This behaviour developed within the first two months and persisted to the third month.

“…but I think I’m quite a stubborn driver so… if someone’s like pulled out on me and gone slow I’m quite happy to drive really close to them (laughs)… erm and I know it’s probably not great but… I guess…when I get a bit annoyed by other drivers… I’m happy to annoy them back (laughs).” Participant 1 Interview 2

Some of the drivers described a thrilling aspect to driving fast as a temptation to resist. The temptation had worn off as the novelty of driving diminished.

“[On] country roads I’ve never gone over but I do like the ability to be able to drive cos… its like you’ve got no limitations and it is an adrenalin… but… when it comes to the point where it becomes a hazard…that’s when it becomes more of a panic situation…” Participant 11 Interview 1

“it's not as thrilling anymore….. it was like when I first did it I was like oh I'm driving…. now I'm just like eurghh…got to drive…[….]….there's more of a temptation to speed round corners when you first pass…but now I have no temptation so I just don't I suppose…”

“So what's the temptation when you first pass do you think?” (Interviewer)

“…thrill…. kind of like a rollercoaster… like thrill-seeking… it's kind of oooh that was fun…like ooh I wonder if my car can manage this grip…” Participant 10 Interview 3

Some of the participants reported difficulties driving with peer passengers. This was particularly problematic when driving at night with passengers who had consumed alcohol. In their early weeks of driving, new drivers found this distracting and difficult to manage. A few responded to peer pressure by taking risks:

“Do you ever feel like you show off when you’re driving?” (Interviewer)

“To my friends yeah….yeah erm….and like braking later just to show that I can…I mean I’ve never thought about it like this but it…probably is that…erm…and….I don’t like sticking to the speed limit when they’re in the car either… At first erm….because they all knew I was a new driver …they were a lot more happy just to like…let me…drive…but now they… they kind of think that…I should be getting used to driving a bit faster…erm or like pushing it a bit more with lights and stuff…” Participant 1 Interview 3

Many of the drivers developed methods to cope with difficult passengers. These involved reprimanding passengers or avoiding giving them lifts altogether.

“Yeah it was the people that I had in the car … when he was drunk he would tell me when to change gear and was like offering to change it for me … which annoyed me, I pulled over to try and get him out at one point.” Participant 7 Interview 1

All of the drivers who had telematic devices felt that they restricted their speed to keep within the speed limit. Most of these drivers reported that this also limited their ability to keep up with traffic flow at first. However, over time they found the device did not register every time they went over the limit and so tended to drive a little faster. They also became more positive about the role of the device in teaching them to be safer drivers.

“Yeah there weren’t a point where I didn't have the black box, it, I mean that keeps you grounded as well I guess, obviously you don’t speed when you’re with instructor anyway else you’ll fail or whatever but it keeps you grounded and it makes you feel like well I’ve got to drive safe to bring my cost down so it gives an incentive.” Participant 9 Interview 1

### 3.3 Social status and pressure

The participants reported that driving gave them a sense of independence and maturity, and enhanced their status relative to their peers:

“…it’s a lot more freedom like… I’ve bought a car, got insurance and everything and just being able to park it at work and leave it there and then drive myself home at night…. it feels so much more like grown up than I did before.” Participant 1 Interview 1

Being seen as a good driver by their peers was important throughout the first three months of driving. Most of the drivers described how their confidence had been affected either by praise or criticism from passengers. Initially many participants felt out of place on the road and they all felt that other drivers were judging them.

“… I also feel like the people around me are thinking "how did she pass her test if she's driving this slowly?"…. so I’m trying to keep up a little bit." Participant 3 Interview 1

All of the drivers felt pressure to drive faster or pull out of junctions quicker from cars behind them. Many mentioned difficulties matching the traffic speed and worried other drivers were annoyed with them for failing to keep up. This was regardless of whether annoyance was shown to them or not. Most reported driving faster in some situations as a result.

“…whenever I’ve got someone behind me I do feel a bit like ‘okay I’ve got to be going at the very speed limit otherwise they’ll be getting really upset about it (laughs).” Participant 3 Interview 1

A number of drivers used ‘P’ (provisional) plates to indicate they were inexperienced or to change the behaviour of other cars around them.

“I have noticed I’ve had a couple of drives without my P Plates on and people overtake me a lot more harshly and are like cutting in front of me or they will stop a lot closer behind me at lights erm but with them on they’ll leave me enough space like if I roll back or something.” Participant 1 Interview 1

By the end of the third month all but one of the drivers reported feeling more secure in their driving abilities and that they fitted in on the road. Consequently, they felt less pressure from other drivers. In some cases, the drivers believed their prior projections of annoyance had been unrealistic:

“I think at the start it were just me making things up. But you do, when you are like … because you don’t feel confident in yourself, so you are like oh my God they are looking at me, they are doing this, they are doing that! But actually they’re not.” Participant 6 Interview 3

## 4 Discussion

This study examined the development of driving behaviour over the first three months of independent driving. We focused on elucidating the behavioural mechanisms underlying the reduction in crash involvement over this time period. Our study had a number of strengths for this purpose. First, the semi-structured qualitative interview supported both a deductive and inductive approach to exploration. Deductive issues were addressed by probing critical issues and situations identified as important in novice driver crash involvement in the existing literature. Inductive advances were facilitated through open ended probes that allowed participants to bring up novel concepts that may have been missed in the existing literature. Second, interviews were collected over repeated contacts spanning the first three months of driving. This allowed participants to report on the process of change as it happened, rather than relying on retrospective report that may be more vulnerable to recall biases. The results yielded are compatible with a number of existing theoretical debates, as described below, and indicate ways to take these debates forward. The interviews also generated new leads that we believe are worthy of further research, as discussed below.

The results must, however, be interpreted in the context of some limitations. First, whilst our detailed analyses were conducted with 36 interviews from 13 participants, it was nevertheless based on data obtained from a volunteer sample recruited from UK educational institutions. As such, the results may not be transferable beyond the sampled population. Given our findings about social pressures, replication with samples drawn from other cultures and settings is particularly important. Despite these limitations, our focused and repeated sampling strategy enabled an in-depth analysis of the process of change over time. In addition, the sample size is commensurate with other thematic analyses. Saturation was achieved as demonstrated by the consistency of the themes generated between participants and the absence of new themes generated by the final participants interviewed.

### 4.1 Driving skills

We found that our participants perceived that their driving skills improved substantially over the first three months of driving. This included vehicle control skills, such as steering, gear-changing, simple road positioning and awareness of their car’s spatial dynamics. As noted in the introduction, we are unaware of any studies that have measured the development of skills of this sort through the driver training and early independent driving phases. Our participants’ beliefs that their skills were continuing to improve during the post-license phase emphasizes the need for studies of this sort. Our participants described simple car control skills as becoming smoother and less attention-demanding as experience was gained; the hallmarks of automaticity (Logan, 1988). These findings imply that simple driving skills required by novice drivers may not have reached an optimal level of automaticity by the end of training. Further automation may support the decrease in crash involvement observed during the first few months of driving.

At least two mechanisms might underlie a lack of automaticity in these driving skills. First, it may be that the learning phase does not provide a sufficient quantity of practice for automaticity to develop. The Cohort II study provides equivocal evidence. Longer periods of training were associated with lower risk of crash involvement but there was no link between amount of either professional training or informal practice and crash involvement (Wells et al., 2008). Further research is required on this issue, ideally taking into account the possibility that inherently safer drivers may choose longer training periods. If it is found that longer training periods are beneficial then it would be possible to specify a minimum number of hours of driving that must be completed before taking a driving test.

A second possibility is that current training does not provide opportunities to practice, and therefore automate, all the critical aspects of skilled driving that are required during independent driving. This was suggested by our participants, who noted that making decisions when supervised differed from making decisions when driving alone and that they found novel situations particularly difficult. The participants also remarked that their post-license learning involved an element of “trial-and-error”, potentially a particularly dangerous form of learning. The general psychology literature shows that transfer is often most effective when training and performance situations are consistent (Barnett and Ceci, 2002). Therefore, research might explore how the training situation can be made more similar to independent driving. Revisions to the UK practical driving test have started to address the issue of independent driving by requiring candidates to follow a route with only satellite-navigation guidance (Helman et al., 2010). With the increasing availability of cheap technology, training might additionally start to include elements of independent driving via simulations.

The participants highlighted an additional transfer challenge when their independent driving began in an unfamiliar car: the need to re-learn aspects of car control. Further research could address the utility of requiring novice drivers to receive a certain number of professional lessons when they begin using an unfamiliar vehicle, or whether requiring people to drive several different types of car while training might help to develop generalizable skills.

Our participants also perceived an improvement in their situation awareness in terms of their understanding of the complex road environment and the behaviour of other drivers. They felt their skills had improved sufficiently to support anticipation of the actions of other drivers and future road states; the highest level of situation awareness according to Endsley (1995). While it is likely that substantial further developments in situation awareness take place across years of practice (Horswill, 2016), any improvements in situation awareness over the first three months of driving might contribute to the reduction in crash liability during this period.

It is commonly argued that effective hazard perception, the anticipation of upcoming dangerous traffic situations, depends on the development of the cognitive skills required to maintain accurate situation awareness (Horswill, 2016). Therefore, that our participants believed that their situation awareness improved may be at odds with the single study that found that hazard perception scores did not differ substantially across this period (Sagberg and Bjornskau, 2006). One possibility is that the Sagberg and Bjornskau study did not have sufficient power to detect change over time. Another possibility, as noted by Sagberg and Bjornskau (2006), and raised by our participants, is that automation of control skills may free attentional resources so that they can be invested in maintaining situation awareness. Simulation measures would not be sensitive to such improvements as they measure hazard perception in isolation from car control. A more powerful and realistic exploration of the time course of novice drivers’ development of situation awareness ability is warranted. This discussion also provides further impetus to explore the benefits of studying the automation of car control skills across driving development.

### 4.2 Violations and thrill-seeking

Consistent with a number of other studies (e.g., Roman et al., 2015), participants reported an overall tendency to drive faster and more aggressively with increasing experience. As noted in the introduction, this tendency runs counter to the decrease in crash involvement observed during this period. Our in-depth exploration provided some pointers to how these opposing trends can co-exist. As with the car control skills, some drivers reported experimenting with their car’s capabilities, particularly when going fast round corners. At some stages this experimentation is likely to provide feedback that the corner has been taken too fast, possibly in the form of a near-crash and occasionally in the form of a crash. This feedback is likely to encourage some reduction in risk-taking, at least in those specific circumstances. A few participants noted that speed was thrilling in the early months of driving but that this diminished over time. This raises the possibility that driving for thrills contributes to the high crash rate in the early weeks of driving.

Further work needs to address both the issues of testing out car capabilities and thrill-seeking during the early months of driving. If these are identified as important aspects of the high crash involvement of newly qualified drivers then the need for prevention efforts will be further emphasized. These aspects of driving may be efficiently combated through enforcement. Telematic devices that monitor driving behaviour offer one option. Our participants that used devices linked to their insurance reported that they did restrict their speed choices, especially during the earliest weeks of driving. Other forms of enforcement that may be able to reduce these forms of behaviour include Graduated Licensing Schemes (GDL) that prohibit novice drivers from driving in situations where thrill-seeking and limit testing is most likely, including driving at night and with same age peers. Schemes of this sort have been implemented in some countries and have been shown to reduce traffic casualties (Williams, 2007).

Our participants perceived pressure to take risks from passengers and there were some reports of responding to these pressures. Although one participant described the pressure as increasing over time, some participants reported that they were finding ways to manage their passengers so that their driving was not compromised; this involved both strategies to manage passengers within the car and strategies to avoid driving with disruptive passengers. The presence of same-age passengers is a well-documented component of risky driving as shown in qualitative (Ehsani et al., 2015; Scott-Parker et al., 2012) and quantitative studies (Ouimet et al., 2015). The evidence from our participants indicates that peers might be most problematic in the earliest stages of driving, before coping strategies have developed and car control skills have become automatic, which might reduce any potential negative impact from peer distraction. Therefore, it is possible that for some drivers, peer effects might contribute to the decrease in crash involvement observed during the first few months of driving, although this will need to be confirmed in further research. As well as the GDL enforcement approach to combating peer influences, educational courses during the learner phase might be able to teach strategies to cope with passengers that could be applied as soon as independent driving begins.

### 4.3 Social status

Participants emphasized the importance of driving to their self-esteem, both in terms of being able to drive and in being perceived as a good driver during real-time driving. The importance of status remained throughout the period studied, but there were substantial developments in the way the participants believed that they were perceived. Initially participants felt that they were inferior to other drivers; they felt unable to keep up with traffic flow and that other drivers perceived them as incompetent. This led to them feeling pressure to drive faster than they would have preferred and to accept gaps that were smaller than they would otherwise have chosen. Over the three months of the study, the participants reported feeling much less pressure from other drivers, citing both their own improving skills and that their original concerns were unrepresentative of the opinions of other drivers. These results are consistent with the findings of Fleiter et al. (2010) which identified perceived pressure from other drivers as a potential risk for dangerous driving in a qualitative study. Unlike our study, Fleiter et al. found that these feelings persisted across a wide age range (17–77 years). However, Fleiter et al. did not focus on very new drivers in their study, so it is possible that these pressures are particularly acute in the early stages of driving.

The desire for status may be a fundamental motivation for many sorts of behaviour in general contexts including displays of overconfidence and conspicuous consumption (Anderson et al., 2015). Little attention has been paid to the concept regarding driving safety. Our results imply that further research is warranted; the participants reported pressure to take risks in the early weeks of driving which reduced over later months. Therefore, behaviour related to status may contribute to the high crash involvement of newly qualified drivers. If this mechanism does prove to be important, then one potential method of remediation might be to make P plates mandatory for the first year of driving. While there is evidence that learner plates increase anger in other drivers (Stephens and Groeger, 2014), our participants who used P plates reported that it made them feel less pressure from other drivers and that other drivers gave them more space as a result. It is also possible that educating learners to drive to their own ability rather than to the perceived expectations of other drivers could be beneficial. This resonates with the Goals for Driver Education (GDE) framework’s recommendation that driver training should aim to better address factors such as personal values, self-control and social context in order to develop safer driving behaviours (Hatakka et al., 2002).

## 5 Conclusion

This work highlights a number of hypotheses regarding behavioural developments that might underlie the safety improvements observed over the first three months of driving. It is likely that more than one developmental process contributes to the decrease in crash involvement, and these processes may be related. For example, as discussed above, automation of vehicle control skills may improve safety in itself, and also increase attentional resources available to support situation awareness. Novices may be more likely to take risks in the very early stage of independent driving at a time when their vehicle control skills are not fully developed, leading to an elevated crash risk. However, crash risk may be lessened both by subsequent improvements in driving skill and reductions in thrill seeking. This might be evident in speeding for example. Although drivers tend to increase speed with experience over the first few months of driving, they may become better able to identify critical situations where speeding would be very dangerous and moderate their speed in these situations. Finally, with regard to social status, perceived pressure from other drivers in the early stages of independent driving might also compound deficits in driving skill, and lead to increased crash risk through novices’ attempts to keep up with the traffic flow, or drive faster in situation they are not fully prepared for. Identifying these processes provides the opportunity to implement interventions and legislation that could substantially reduce the over-involvement of newly qualified drivers in road traffic crashes. Additionally the finding that driving skills may not be fully developed during the early independent driving phase provides additional support for GDL schemes as a protective mechanism to allow skills to develop in lower risk environments. Implications for alternative, non-legislative approaches to novice driver safety include a need for pre- and post-licence training to focus more on risk and risk situations, social status and personal self-control, in line with objectives of the GDE framework.

## Footnotes

1 Stalling is a relatively common problem in the UK where the majority of cars have manual transmission.

Appendix A Supplementary material related to this article can be found, in the online version, at doi:https://doi.org/10.1016/j.aap.2018.04.007.
