# Supplementary material for: Why do drivers become safer over the first three months of driving? A longitudinal qualitative study
Source: Accid Anal Prev. 2018 Aug;117:225–31. doi: 10.1016/j.aap.2018.04.007 (PMC6004036; doi:10.1016/j.aap.2018.04.007)
Supplement: Supplementary file 1 [file mmc1.docx]

**Supplementary information (for on-line publication)**

Interview Schedule

Time 1:

*Preamble:*

‘Thank you for taking part in this study. We are interested in understanding your experiences of driving and how your approach to driving has changed since you passed your test. In order to see how things continue to change over the first few months of driving, we’d like to meet with you again on two additional occasions over the next three months.

In order for our study to deliver useful information we’re keen for you to be really open with us about how you actually drive (rather than how you think others want you to drive!). As it says in the information sheet and consent form, anything you tell us will be used anonymously; we will not share your information with anyone else in any way that could identify you. We would like to use parts of what you tell us in presentations, publications, and for teaching. We will also use the information to inform future driver training.

If at any point you’d like to stop the interview or ask further questions just let me know. Do you have any questions that you’d like to ask before we begin?’

Time 2 and 3

*Preamble:*

‘Thank you for continuing to take part in this study. As you are aware we are interested in finding out today how your driving might have changed over the month since I last saw you.

Just like before, in order for our study to be successful and deliver useful information, it is important that you’re as open and honest with us about your driving experience as possible. As we said before what you choose to tell us will only be used anonymously.

If at any point you’d like to stop the interview or ask further questions just let me know. Do you have any questions that you’d like to ask before we begin?’

**Question areas:**

*Reminder for the interviewer: The interview should follow a ‘critical incident’ approach. In this context* ***critical incidents are instances of positive change/improvement that occur in driving behaviour.*** *We are particularly interested in discovering how such improvements come about in the situations known to be associated with risk (turning across traffic, night driving, driving too close, taking corners/bends and speed choice). The interview needs to focus on how behaviour changed in the key situations so as to elicit information that can be translated into the questionnaire.*

*Particular attention should be paid to gaining information as to participants’ behaviours/actions taken during the change situation and any influencing factors that have had an effect on their subsequent driving behaviour.*

Follow up on references to change- what, where, how, why, context.

How does it feel? Eg. excitement, fear etc. Thoughts around change.

**Current general experience of car use: Remember to ask about the run up to any instances of positive change; gain a full account of the course of events; and the outcome. You should also gain an account of the participants’ behaviours/actions taken; and influence (if any) on driving behaviour. Also look out for the role played by other people who might be in the car.**

1. ‘How did you find driving when you were learning?’ (for follow-up interviews begin with question 2).

Prompts: ‘What was it like for you when learning?’; ‘How did having the instructor in the car affect how you drove?

1. ‘How do you find driving now in comparison to when you were learning/since we last spoke?’

Prompts: ‘What’s it like to drive without supervision?’

“How has your driving changed since you were a learner/last time we met?”

‘What else has changed since passing your test/last time?

1. ‘How have your driving skills improved since you passed your test/ over the last month?

Prompts: ‘Is there anything you have got better at in your driving’

Are there any things about driving you find particularly difficult? Do you think you are getting better at them?

1. Do you think the way you drive has changed? If so, could you describe how?

Prompts: Do you drive faster/slower/ more carefully/ less carefully etc.

Are there any incidents while driving which have changed how you drive?

Possible prompt questions: (only to be used where questions 1-4 don’t elicit discussion)

‘What do you enjoy most about driving since passing your test/since last time we spoke’ ‘Do you enjoy driving and if so what do you like most’

Prompts: Refer back to the situations described in the demographic questionnaire and ask for example ‘so you drive socially, what do you enjoy about this?’ etc.

‘What do you dislike most about driving since passing your test/since last time?’ ‘Is there anything you don’t like about driving? Has this changed since last time we spoke?

Prompts: Refer back to the situations described in the demographic questionnaire and ask for example ‘so you drive socially, what do you dislike about this?’ etc.

‘What do you find most difficult about driving since passing your test/since last time?’ Is there anything you find difficult about driving?

Prompts: ‘Are there particular roads/situations that cause you concern/worry?’

**Use of speed:**

1. ‘What sorts of things affect what speed you drive at now?’ Prompts: importance of speed limit, purpose of journey (eg driving for pleasure); things in the car (eg music?); mood; other passengers/drivers etc.
2. ‘How has the speed you drive altered since you passed your test/since last time we spoke?’ Follow up on change: what, how, why, context, feelings/thoughts around change etc.
3. ‘Are there any situations/occasions now where you take more care about how fast you’re going?’ Prompts: situations; things in the car; mood; other people etc.

**Close following:**

1. ‘How close do you tend to drive to cars in front of you?’
2. Has this changed since your test/last time we spoke? ‘Do you think you drive closer or further away from the cars in front of you since passing your test/ since we last spoke? Follow up on change: what, how, why, context, feelings/thoughts around change etc.
3. ‘How do you decide how close to drive?’ ‘What things affect how close you drive to other cars?’ Prompts: ‘any situations; things in the car; mood; other people (passengers/other driver factors) etc that influence this?’ ‘Are there situations where you deliberately keep a distance?’ Are there times where you find yourself getting too close to the car in front? Have there been any incidents or near incidents associated with close following- only if brought up by interviewee?

**Control on curves/bends:**

1. ‘How do you find driving on sharp bends like the ones often found on country roads?’ Please tell me about the last time you were driving round a tight bend? Can you talk me through your approach?
2. ‘Any changes in your approach to this sort of road since qualifying’ (for follow-up ask about change since last interview)?’ Follow up on change: what, how, why, context, feelings/thoughts around change etc.
3. What influences how fast you go around bends? Prompt: ‘any times that you have nearly lost control’ if yes, has this improved how you approach this situation now? Are there things about driving that make you go faster round bends eg. the thrill of it, passengers in the car, listening to music…

**Turning right across the path of other drivers: (may have to demonstrate this situation using props)**

1. ‘How do you find turning right across the path of other drivers?’ Prompts: for accounts of turning, how do you approach these situations? What things do you look out for? Have you ever had any difficulties with this situation?
2. ‘Any changes in your approach since your test/since we last spoke?’ Follow up on change: what, how, why, context, feelings/thoughts around change etc.
3. “How do you find it when other people turn right in front of you? Prompts: What do you do when you see someone waiting? Have you ever had someone cross right in front of you?

**Driving at night:**

1. ‘How do you find driving at night?’ Prompts: Any different from the daytime? What do you differently? Any differences in reasons for driving in day/night? Are there any other factors that affect how you drive (eg. passengers, music etc.) Have you had any incidents involved with night time driving?
2. ‘Any changes in how you drive at night since qualifying/ last time we spoke? Follow up on change: what, how, why, context, feelings/thoughts around change etc.

**Final question:**

We have asked you about close following of other cars, driving round sharp bends, turning right across traffic and driving at night because they are aspects of driving that can cause problems for new drivers. Are there any other situations you think we should know about? Prompts: areas of driving you find difficult? Any aspect of driving you feel you have improved at since your test/ last time we spoke. Follow up on change: what, how, why, context, feelings/thoughts around change etc.

Thank you, do you have any questions.
